# Sleep duration and cause-specific mortality among US adults with heart disease: A NHIS-NDI linkage cohort study

**DOI:** 10.1371/journal.pone.0362657

**Published:** 2026-11-13

**Authors:** Tolulope Victoria Adebile, Tobi Oloyede, Emmanuel Azu, Mobolaji Olagunju, Olamide Asifat, Temitayo Mary Paul, Oluwaseun Ipede, Wandoo Abwa, Purbasha Biswas, Lili Yu

**Affiliations:** 1 Department of Biostatistics, Epidemiology and Environmental Health Sciences, Jiann-Ping Hsu College of Public Health, Georgia Southern University, Statesboro, Georgia, United States of America; 2 Department of Public Health Sciences, The Pennsylvania State University College of Medicine, Hershey, Pennsylvania, United States of America; 3 Department of Health Policy and Community Health, Jiann-Ping Hsu College of Public Health, Georgia Southern University, Statesboro, Georgia, United States of America; 4 Epidemiology and Biostatistics Department, School of Public Health, Nanjing Medical University, Nanjing, China; 5 Internal Medicine, Mercy One Hospital, Des Monies, Iowa, United States of America; 6 Department of Geology and Geography, Georgia Southern University, Statesboro, Georgia, United States of America; National Yang Ming Chiao Tung University School of Medicine, TAIWAN

## Abstract

**Background:**

Sleep duration is increasingly recognized as a component of cardiovascular health, yet its prognostic significance among adults with established heart disease remains unclear. We examined associations between sleep duration and mortality among US adults with heart disease.

**Methods:**

We analyzed data from 3,287 adults with heart disease participating in the 2004 National Health Interview Survey linked to the National Death Index through December 31, 2019. Sleep duration was categorized as ≤6 hours, 7–8 hours, and ≥9 hours. Cox proportional hazards models stratified by variables violating the proportional hazards assumption estimated hazard ratios (HRs) and 95% confidence intervals (CIs) for all-cause, heart-related, and non-heart-related mortality. Restricted cubic splines evaluated nonlinear dose-response relationships.

**Results:**

During 15 years of follow-up, 49.4% of participants died, including 16.7% from heart-related causes and 32.6% from non-heart-related causes. Compared with 7–8 hours of sleep, long sleep duration (≥9 hours) was associated with higher risks of all-cause mortality (HR:1.51 [95% CI:1.32–1.73]), heart-related mortality (HR:1.53 [95% CI:1.22–1.92]), and non-heart-related mortality (HR:1.50 [95% CI:1.27–1.77]). Restricted cubic spline analyses demonstrated nonlinear associations, with the lowest predicted mortality risk occurring at 6–7 hours. Findings remained robust across multiple sensitivity analyses and age and sex subgroups.

**Conclusions:**

Among US adults with heart disease, long sleep duration was consistently associated with higher risks of all-cause, heart-related, and non-heart-related mortality. Sleep duration may represent a simple marker of residual risk that could enhance risk stratification among adults living with heart disease.

## Introduction

Heart disease remains the leading cause of death in the United States (US), contributing substantially to premature mortality, disability, and health care costs despite major advances in prevention and treatment [1,2]. As survival improves, a growing number of adults are living with a history of heart disease and remain vulnerable to recurrent events, functional decline, multimorbidity, and premature death [1,3]. Historically, clinical management has focused on controlling traditional cardiovascular risk factors such as hypertension, diabetes, dyslipidemia, and smoking. However, growing recognition that these factors alone do not fully explain long-term outcomes among this high-risk population has prompted a broader shift toward lifestyle-based approaches to secondary prevention and risk stratification, focusing on modifiable health behaviors [1].

A particularly attractive candidate for lifestyle prescription is sleep duration because it is low-cost, scalable, routinely assessable, and potentially modifiable [4,5]. Sleep plays a critical role in metabolic regulation, inflammation, autonomic function, and cardiovascular recovery, and accumulating evidence links both short and long sleep duration with adverse health outcomes, including incident cardiovascular diseases and premature mortality in the general population [4–10]. Long sleep duration has also been associated with increased mortality among individuals with chronic noncommunicable disease populations [11], while healthier multidimensional sleep patterns (e.g., adequate sleep duration) have been associated with lower risks of cardiovascular diseases and all-cause mortality among adults with diabetes [12].

Adults living with heart disease remain at elevated risk of premature mortality despite substantial advances in cardiovascular prevention and treatment. Identifying simple and scalable markers of residual risk is therefore an important priority for both clinical care and population health. Sleep duration is increasingly recognized as a component of cardiovascular health, yet its prognostic relevance among individuals with established heart disease remains incompletely understood. In particular, it remains unclear whether sleep duration is associated similarly across all-cause, heart-related, and non-heart-related mortality outcomes and whether these relationships follow the nonlinear patterns observed in the general population and among US patients with chest pain [13,14]. However, evidence remains limited among adults with established heart disease, particularly regarding whether sleep duration is associated similarly with all-cause, heart-related, and non-heart-related mortality outcomes [14,15]. Using nationally representative data from the National Health Interview Survey linked to the National Death Index, we examined associations between sleep duration and all-cause, heart-related, and non-heart-related mortality among US adults with heart disease. We additionally evaluated potential nonlinear dose-response relationships and assessed the robustness of findings through subgroup and sensitivity analyses.

## Materials and methods

### Data source and study population

Data from the 2004 National Health Interview Survey (NHIS), a nationally representative cross-sectional survey of the noninstitutionalized US population conducted by the National Center for Health Statistics (NCHS) was used in this study. The NHIS uses a multistage stratified sampling design to generate population-representative estimates of the United States adult population [16]. Participants were linked to mortality status through December 31, 2019, using the National Death Index Linked Mortality Files. To achieve this, the NCHS uses a probabilistic mortality matching scheme to create the Linked Mortality File [17].

Among 94,597 participants in the 2004 survey cycle, we included those aged 18 years or older with complete data on sleep duration, heart disease, mortality follow-up, and study covariates (n = 3,287). Heart disease (yes, no) was assessed based on the information provided on “Ever been told you had coronary heart disease,” “Ever been told you had a heart condition/disease,” and “Ever been told you had a heart attack.”

These data are publicly available from the Center for Disease Control and Prevention website and have been approved by the NCHS’ ethics review board. There is no human/animal involved in this study.

### Exposure assessment

The primary exposure was self-reported sleep duration, assessed by the question: “On average, how many hours of sleep do you get in a 24-hour period?” Sleep duration was categorized as ≤6 hours (short), 7–8 hours, and ≥9 hours (long) per night, an adaptation of existing recommendations [15,18]. To evaluate potential nonlinear associations and avoid information loss from categorization, secondary analyses modeled sleep duration as a continuous exposure.

### Outcome assessment

The primary outcomes were all-cause, heart disease-related (ICD-10 codes I00–I09, I11, I13, I20–I51), and non-heart disease-related mortality during follow-up. For cause-specific analyses, deaths from causes other than the outcome of interest were censored at time of death. Survival time was calculated from the date of NHIS interview to date of death or administrative censoring on December 31, 2019, whichever occurred first.

### Study covariates

Sociodemographic variables included age (<65 years and ≥65 years), sex, marital status (married, single/never married, and separated/divorced/widowed), and poverty-to-income ratio (< 2.0, 2.00–4.99, > 4.99). Health behavior variables included smoking and drinking status (current, former, never), physical activity (light/moderate, never/unable), and body mass index (BMI, < 25 kg/m^2^, 25- ≤ 30 kg/m^2^, > 30 kg/m^2^). Clinical variables included self-reported hypertension, diabetes, stroke, and emphysema. Covariates were selected a priori based on established literature and their clinical relevance as potential confounders of the association between sleep duration and mortality.

### Statistical analysis

The SAS programming software (version 9.4; English) was used for all analysis in this study. Participant characteristics were described overall and by subgroups (age and sex) using means with standard deviations for continuous variables and frequencies with percentages for categorical variables. Differences across groups were evaluated using chi-square tests for categorical variables and appropriate tests for continuous variables.

Kaplan–Meier survival curves were used to compare cumulative survival across sleep-duration categories, with differences assessed using the log-rank test.

Cox proportional hazards regression models were used to estimate hazard ratios (HRs) and 95% confidence intervals (CIs) for the association between sleep duration and mortality. The proportional hazard assumption was tested using time-dependent covariate interactions, and violators (BMI, physical activity, and stroke) were incorporated through stratified Cox models to allow separate baseline hazards. Sequential models were fitted to evaluate robustness of associations: Model 1 adjusted for demographic factors; Model 2 additionally adjusted for health behavioral factors; and Model 3 further adjusted for clinical comorbidities. Models 2 and 3 were stratified models, addressing violators (BMI, physical activity, and stroke) of proportional hazard assumptions. To examine potential nonlinear dose-response associations between continuous sleep duration and mortality risk, the fully adjusted stratified Cox restricted cubic spline model was employed. Where a nonlinear relationship was found, the threshold point was estimated and further explored.

To assess whether associations differed across subgroups (age and sex), multiplicative interaction terms of sleep duration with age and sex were tested as a secondary analyses, and subgroup differences reported.

To evaluate the robustness of our findings, ten sensitivity analyses were conducted using Model 3. First, to assess potential confounding by digestive-related health problems, we additionally adjusted for digestive-related functional limitation (self-reported digestive system problem causing difficulty with activities). Second, to reduce potential reverse causation from underlying illness that may have influenced sleep duration and mortality risk, we repeated analyses after excluding participants who died within the first two years of follow-up. Third, because heart disease was based on self-reported history and may represent a heterogeneous clinical population, we repeated the analyses separately among participants reporting coronary heart disease (CHD), myocardial infarction (MI), and any other heart disease/condition. Although angina was not used to define the primary analytic population, we also conducted a sensitivity analysis among participants with self-reported angina to evaluate whether findings were consistent in this clinically relevant subgroup. Fourth, for heart-related and non-heart-related mortality, we estimated subdistribution hazard ratios using Fine–Gray competing-risk models, treating deaths from other causes as competing events. Fifth, we redefined heart disease-related mortality to include deaths from cerebrovascular diseases (I60-I69), reflecting a broader cardiovascular mortality definition. Sixth, we accounted for NHIS sampling weights and variance-estimation variables to assess whether findings were robust to the complex survey design and generalizability to the US adult population. Finally, because cancer history is strongly associated with mortality risk and may also influence sleep duration, we repeated the analyses after excluding participants with a self-reported history of cancer.

Findings from these sensitivity analyses were compared with the primary fully adjusted Cox proportional hazards models (Model 3) to assess consistency in the direction, magnitude, and statistical significance of the associations between sleep duration and mortality. Statistical significance was defined as a 2-sided P value <0.05.

## Results

### Population characteristics

Table 1 presents the characteristics of the study population (n = 3,287). Participants characteristics differed significantly across age (< 65 years vs. ≥ 65 years) and sex (male vs. female), except for stroke, emphysema, hypertension, and physical activity, which did not differ significantly by sex.

**Table 1:** Characteristics of participants with heart disease (N = 3,287).

		Age [N (%)]		P-value	Sex [N (%)]		P-value
	Overall	< 65 years	≥ 65 years		Male	Female	
**Os (mean ± std)**	137.0 ± 60.3	165.5 ± 42.3	107.3 ± 7.5	<.0001	131.8 ± 61.1	141.1 ± 59.3	<.0001
**Sleep duration (mean ± std)**	7.2 ± 1.7	6.9 ± 1.6	7.5 ± 1.7	<.0001	7.3 ± 1.7	7.1 ± 1.7	0.0030
**Marital status**				<.0001			<.0001
Married	1560 (47.5)	876 (56.2)	684 (43.9)		875 (56.1)	685 (43.9)	
Separated/ divorce/ widowed	1347 (41.0)	490 (36.4)	857 (63.6)		413 (30.7)	934 (69.3)	
Single/ never married	380 (11.6)	308 (81.1)	72 (18.9)		173 (45.5)	207 (54.5)	
**Smoking**				<.0001			<.0001
Current	593 (18.0)	463 (78.1)	130 (21.9)		278 (46.9)	315 (53.1)	
Former	1210 (36.8)	449 (37.1)	761 (63.9)		705 (58.3)	505 (41.7)	
Never	1484 (45.2)	762 (51.4)	722 (48.7)		478 (32.2)	1006 (67.8)	
**Drinking**				<.0001			<.0001
Current	833 (25.3)	338 (40.6)	495 (59.4)		230 (27.6)	603 (72.4)	
Former	867 (26.4)	376 (43.4)	491 (56.6)		436 (50.3)	431 (49.7)	
Never	1587 (48.3)	960 (60.5)	627 (39.5)		795 (50.1)	792 (49.9)	
**Physical activity**				<.0001			0.0811
Light/moderate	1481 (45.1)	843 (56.9)	638 (43.1)		683 (46.1)	798 (53.9)	
Never/unable	1806 (54.9)	831 (46.0)	975 (54.0)		778 (43.1)	1028 (56.9)	
**BMI**				<.0001			<.0001
Normal/ Underweight	1137 (34.6)	539 (47.4)	598 (52.6)		415 (36.5)	722 (63.5)	
Overweight	1135 (34.5)	528 (46.5)	607 (53.5)		640 (56.4)	495 (43.6)	
Obese	1015 (30.9)	607 (59.8)	408 (40.2)		406 (40.0)	609 (60.0)	
**Diabetes**				<.0001			0.0007
Yes	656 (20.0)	266 (40.6)	390 (59.4)		329 (50.2)	327 (49.9)	
No	2555 (77.7)	1379 (54.0)	1176 (46.0)		1091 (42.7)	1464 (57.3)	
Borderline	76 (2.3)	29 (38.2)	47 (61.8)		41 (54.0)	35 (46.0)	
**Hypertension**	1908 (58.1)	799 (41.9)	1109 (58.1)	<.0001	855 (44.8)	1053 (55.2)	0.6218
**Stroke**	412 (12.5)	150 (36.4)	262 (63.6)	<.0001	182 (44.2)	230 (55.8)	0.9051
**Emphysema**	219 (6.7)	88 (40.2)	131 (59.8)	0.0010	108 (49.3)	111 (50.7)	0.1335
**Sleep Duration**				<.0001			0.0042
≤6 hours	1075 (32.7)	647 (60.2)	428 (39.8)		436 (40.6)	639 (59.4)	
7-8 hours	1774 (54.0)	874 (49.3)	900 (50.7)		832 (46.9)	942 (53.1)	
≥9 hours	438 (13.3)	153 (34.9)	285 (65.1)		193 (44.1)	245 (55.9)	
**Mortality**							
All-cause	1623 (49.4)	378 (22.3)	1245 (76.7)	<.0001	797 (49.1)	826 (50.9)	<.0001
Heart-related	550 (16.7)	125 (22.7)	425 (77.3)	<.0001	285 (51.8)	265 (48.2)	0.0001
Non-Heart-related	1073 (32.6)	253 (23.6)	820 (76.4)	<.0001	512 (47.7)	561 (52.3)	0.0086

OS = overall survival in months. P-values were calculated using the chi-square test for categorical variables and analysis of variance (ANOVA) for continuous variables.

The mean (SD) overall survival was 137 (60.3) months and was highest among participants aged < 65 years and among females. The mean sleep duration in the study population was approximately 7 hours. Participants were more likely to be never smokers, never drinkers, married, physically inactive, non-diabetic, and have a history of hypertension.

Participants < 65 years had a mean sleep duration of approximately 7 hours and were more likely to be single/never married, never smokers, never drinkers, physically active, and obese. They were also less likely to have hypertension, stroke, or emphysema.

In addition, female participants reported a mean sleep duration of approximately 7 hours. Compared to males, females were more likely to be divorced, have a normal weight/underweight BMI, non-diabetic, and be never smokers, but were more likely to be current drinkers.

The prevalence of all-cause mortality was 49.4%, while heart-related and non-heart-related mortality accounted for 16.7% and 32.6% of deaths, respectively. Mortality rates were higher among participants ≥ 65 years old, and for all heart-related-deaths, rates were highest among males (51.8%).

### Sleep duration and mortality risk

Fig 1 presents the Kaplan-Meier survival curves according to sleep duration categories. Participants reporting long sleep duration (≥ 9 hours) had the lowest survival probabilities throughout follow-up. Survival probabilities differed significantly across sleep duration categories (log rank p < 0.001).

**Fig 1:**
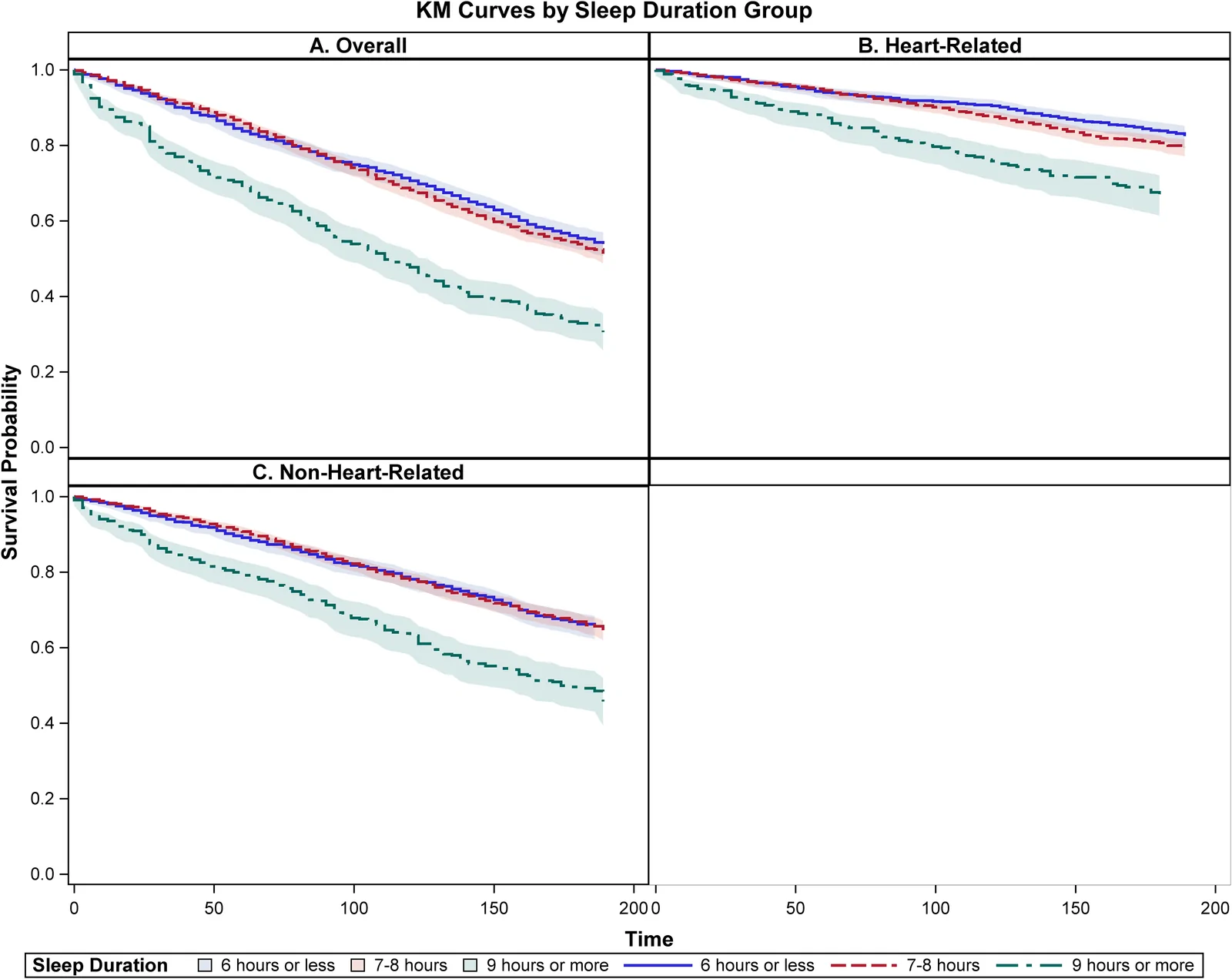
Kaplan Meier Curve with 95% confidence interval for survival probability by sleep duration among individuals with heart disease: A: all-cause mortality, B: heart-related mortality, and C: non-heart related mortality. Log-rank test of group differences showed significant difference by sleep duration categories (p < 0.001).

Participants reporting long sleep duration experienced consistently higher all-cause, heart-related, and non-heart-related mortality throughout follow-up. In the fully adjusted model, long sleep was associated with a 51% higher risk of all-cause mortality (HR: 1.51, 95% CI: 1.32–1.73), a 53% higher risk of heart-related mortality (HR: 1.53, 95% CI: 1.22–1.92), and a 50% higher risk of non-heart-related mortality (HR: 1.50, 95% CI: 1.27–1.77 (Table 2).

**Table 2:** Sleep duration and mortality risk among individuals with heart disease (n = 3287).

	All-Cause			Heart-Related			Non-Heart-Related		
	≤6 hours	7-8 hours	≥ 9 hours	≤6 hours	7-8 hours	≥ 9 hours	≤6 hours	7-8 hours	≥ 9 hours
**Model 1**	1.11 (0.99, 1.24)	1 (ref)	1.66 (1.45, 1.90)	0.97 (0.79, 1.18)	1 (ref)	1.71 (1.37, 2.14)	1.19 (1.03, 1.36)	1 (ref)	1.63 (1.38, 1.93)
**Model 2** ^*^	1.05 (0.94 1.18)	1 (ref)	1.56 (1.37, 1.79)	0.92 (0.75, 1.12)	1 (ref)	1.63 (1.30, 2.03)	1.13 (0.98, 1.29)	1 (ref)	1.53 (1.30, 1.81)
**Model 3** ^*^	1.01 (0.90, 1.13)	1 (ref)	1.51 (1.32, 1.73)	0.88 (0.72, 1.07)	1 (ref)	1.53 (1.22, 1.92)	1.08 (0.94, 1.24)	1 (ref)	1.50 (1.27, 1.77)

**Model 1:** sleep duration, age, sex, marital status. **Model 2:** Model 1 + body mass index (BMI), smoking, drinking, physical activity. **Model 3:** Model 2 + hypertension, diabetes, stroke, and emphysema. Models 2 and 3 are stratified Cox models, addressing violators (BMI, physical activity, and stroke) of proportional hazard assumptions. ^*^Results remained consistent in unstratified models.

Although no statistically significant interactions were observed by age and sex, the association between long sleep duration and mortality was generally consistent across subgroups. The strongest association with heart-related mortality was observed among participants aged ≥ 65 years and among females, whereas the strongest association with non-heart-related deaths was observed among participants aged <65 years and among males (Table 3).

**Table 3:** Sleep duration and mortality risk among individuals with heart disease by age and sex.

	All-Cause			Heart-Related			Non-Heart-Related		
	≤6 hours	7-8 hours	≥ 9 hours	≤6 hours	7-8 hours	≥ 9 hours	≤6 hours	7-8 hours	≥ 9 hours
**Age** ^*^									
<65 years	0.99 (0.79, 1.24)	1 (ref)	1.46 (1.05, 2.01)	0.83 (0.56, 1.22)	1 (ref)	1.17 (0.65, 2.11)	1.09 (0.83, 1.44)	1 (ref)	1.63 (1.11, 2.39)
≥ 65 years	1.02 (0.89, 1.16)	1 (ref)	1.46 (1.26, 1.70)	0.89 (0.70, 1.13)	1 (ref)	1.56 (1.22, 2.00)	1.09 (0.93, 1.28)	1 (ref)	1.41 (1.17, 1.70)
**Sex** ^*^									
Male	1.05 (0.89, 1.24)	1 (ref)	1.62 (1.33, 1.98)	0.82 (0.62, 1.10)	1 (ref)	1.47 (1.06, 2.03)	1.19 (0.97, 1.46)	1 (ref)	1.72 (1.34, 2.20)
Female	0.99 (0.85, 1.16)	1 (ref)	1.42 (1.18, 1.71)	0.92 (0.69, 1.22)	1 (ref)	1.57 (1.14, 2.17)	1.02 (0.84, 1.24)	1 (ref)	1.33 (1.06, 1.68)

Fully adjusted stratified Cox model, including sleep duration, age, sex, marital status, body mass index (BMI), smoking, drinking, physical activity, hypertension, diabetes, stroke, and emphysema. Model was stratified by violators (BMI, physical activity, and stroke) of proportional hazard assumptions. ^*^P-value for test of interaction was not significant (p > 0.05).

In contrast, short sleep duration (≤6 hours) was significantly associated only with non-heart-related mortality in Model 1, and this association was attenuated after adjusting for other covariates (Tables 2 and 3).

Restricted cubic spline analysis demonstrated a significant non-linear U-shape relationship between sleep duration and both all-cause and non-heart-related mortality, with mortality risk increasing markedly at longer sleep durations (Fig 2). Based on these analysis, the estimated sleep durations associated with the lowest mortality risk were 6.5 hours for all-cause mortality, 6 hours for heart-related mortality, and 6.8 hours for non-heart-related mortality.

**Fig 2:**
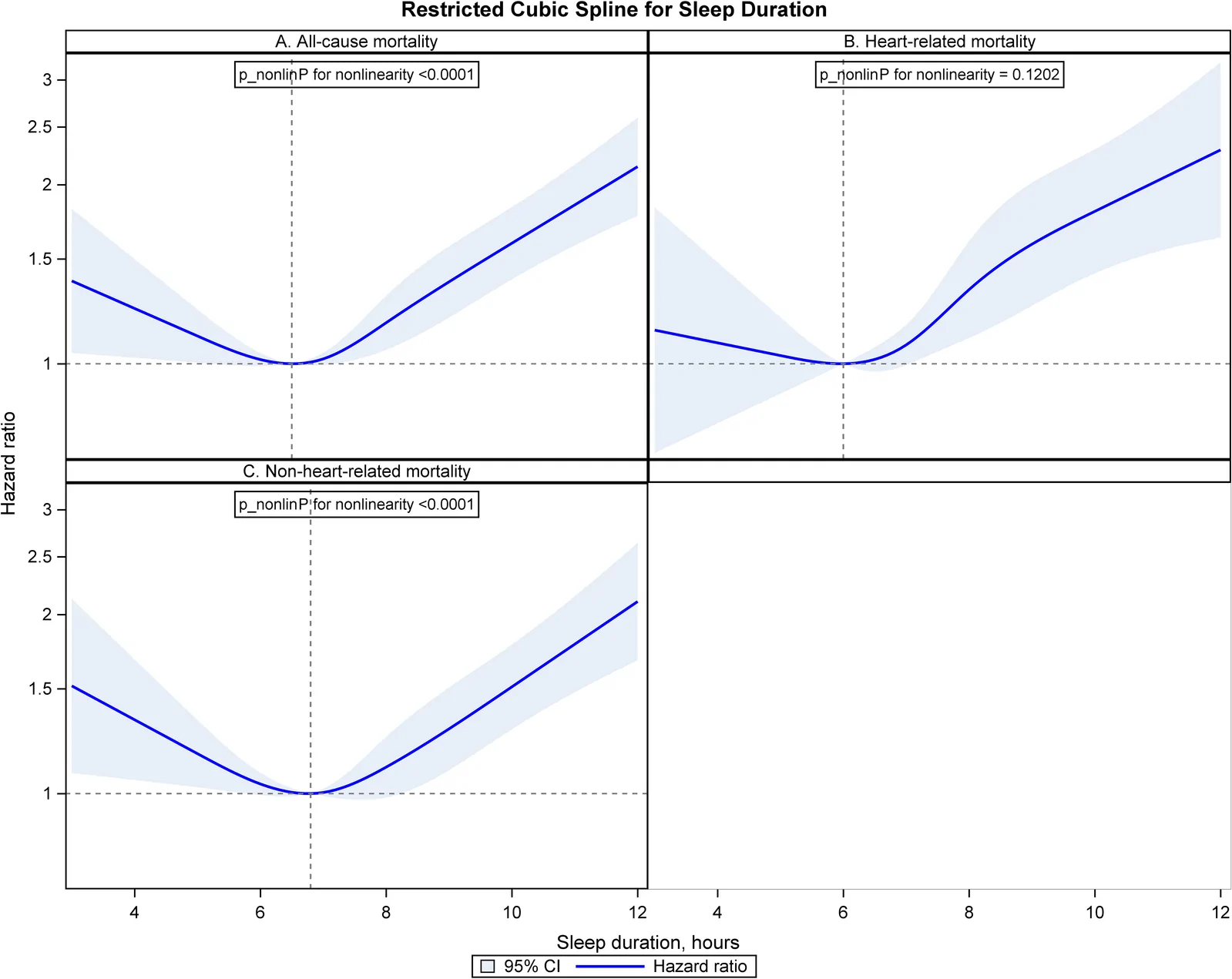
Restricted cubic spline curves with 95% confidence intervals for sleep duration and mortality, with lowest predicted hazard ratio at 6.5, 6, and 6.8 hours for all-cause (A), heart-related (B), and non-heart-related mortality (C), respectively. Nonlinearity was assessed using a Wald test of the spline terms.

### Sensitivity analyses

Findings from the sensitivity analyses were generally consistent with the primary results (S1 Table). Across sensitivity analyses, long sleep duration (≥9 hours) was consistently associated with higher mortality risk compared with 7–8 hours of sleep, whereas short sleep duration (≤6 hours) was generally not associated with all-cause or heart-related mortality.

For all-cause mortality, the hazard ratio for ≥9 hours of sleep ranged from 1.42 to 1.77 across sensitivity analyses. Notably, in the survey-weighted analysis, long sleep duration was associated with a 26% higher risk of all-cause mortality. Similar patterns were observed in analyses excluding cancer survivors and disease-specific subgroups, including CHD, MI, angina, and other heart disease/conditions. Elevated risks were also seen across heart-related and non-heart-related mortality outcomes and other sensitivity analyses.

## Discussion

This nationally representative cohort study examined the association between sleep duration and mortality among US adults living with heart disease. Three principal findings emerged. First, long sleep duration (≥9 hours) was independently associated with substantially higher risks of all-cause, heart-related, and non-heart-related mortality. Second, restricted cubic spline analyses demonstrated nonlinear associations between sleep duration and mortality, with the lowest predicted risk occurring at approximately 6–7 hours of sleep per night. Third, these associations were broadly consistent across age and sex groups and remained robust across multiple sensitivity analyses.

The observed association between long sleep duration and mortality is consistent with growing evidence from chronic disease cohorts, general populations, and symptomatic cardiovascular-risk populations reporting elevated risk at the upper end of the sleep distribution [13–15,19–21]. Compared with 7–8 hours, sleeping ≥9 hours was associated with more than 50% higher risks of all-cause, heart-related, and non-heart-related mortality after adjusting for demographic, behavioral, and clinical factors, suggesting that prolonged sleep may function less as a causal exposure and more as a marker of underlying vulnerability. Notably, the effect sizes in the present study were larger than those reported by Cappuccio et al. in a meta-analysis of 27 prospective cohort samples from the general population, which found a 30% increased risk of all-cause mortality among long sleepers in general-population cohorts [21]. This difference suggests that prolonged sleep may carry stronger prognostic information in clinically vulnerable cardiovascular populations than in the general population. Long sleep duration has been associated with lower sleep efficiency, multimorbidity, depression, chronic inflammation, medication burden, sleep-disordered breathing, low physical functioning, or unmeasured disease severity, all of which may contribute to elevated mortality risk [19,22,23]. In individuals already living with cardiovascular disease, these conditions may be especially relevant because they can signal worsening health status not fully captured by conventional clinical risk factors. As such, our findings support the interpretation of prolonged sleep as a marker of underlying vulnerability and less as a direct causal exposure [24].

The nonlinear U-shaped associations observed for all-cause and non-heart-related mortality, with the highest risks at the longer end of the sleep spectrum, further highlight long sleep duration as a salient prognostic marker. Consistent with previous studies, these findings suggest that mortality risk varies across the sleep-duration spectrum rather than reflecting a simple threshold effect [13,15,21]. The lowest predicted hazard occurring at approximately 6.5 hours for all‑cause mortality and 6.8 hours for non‑heart‑related mortality is consistent with prior meta‑analyses and with current recommendations suggesting that 6–8 hours of sleep may be optimal for individuals with cardiovascular disease [19,20]. For heart-related mortality, the spline analyses suggested a nonsignificant nonlinear association, with the lowest predicted risk occurring at approximately 6 hours and elevated risk at longer sleep durations. Prior evidence from a symptomatic cardiovascular-risk population of U.S. adults with chest pain found increased cardiovascular mortality at both short and long sleep durations, whereas our heart-related mortality analyses suggested risk increased more consistently at longer sleep durations [14]. Despite this difference in curve shape, these findings reinforce long sleep duration as an adverse prognostic marker in cardiovascular-risk populations and suggest that survival among adults with heart disease may be most favorable within a relatively narrow sleep-duration range (6–7 hours).

There was no statistically significant interaction by age or sex for all-cause, heart-related, or non-heart-related mortality, suggesting that the prognostic relevance of sleep duration was broadly shared across demographic groups. Although risks varied slightly between subgroups, elevated risk with long sleep duration was generally observed in both younger (< 65 years) and older (≥ 65 years) adults and in both men and women. For heart-related mortality, however, the association with long sleep duration was weaker and not statistically significant among adults aged <65 years. This pattern may reflect the lower absolute burden of fatal heart-related events in this younger subgroup, resulting in fewer events and limited statistical precision. It is also possible that, among younger adults with heart disease, long sleep duration is more closely tied to non-heart-related pathways, such as underlying illness, occupational or behavioral risks, or other comorbid conditions. In contrast, older adults, may have higher burdens of multimorbidity, frailty, or sleep‑disordered breathing that increase vulnerability to heart‑related deaths. Nevertheless, because tests for interaction were not statistically significant, these subgroup differences should be interpreted cautiously and considered hypothesis-generating.

The nonsignificant association between short sleep duration and non-heart-related mortality after adjusting for BMI, smoking, drinking, physical activity, hypertension, diabetes, stroke, and emphysema suggests that at least part of the relationship between short sleep and mortality may be explained by coexisting behavioral and clinical risk factors. Prior studies have linked insufficient sleep to adverse cardiometabolic outcomes through pathways involving sympathetic activation, impaired glucose metabolism, endothelial dysfunction, and elevated blood pressure [25,26]. Our results do not negate these mechanisms, but they suggest that within a population already living with heart disease, the independent prognostic signal of short sleep may be less pronounced than that of long sleep. Importantly, associations remained robust across all sensitivity analyses, including exclusion of early deaths, restriction to participants with CHD or prior MI, competing-risk models, and survey-weighted models accounting for the NHIS complex sampling design. These findings suggest that the observed relationships were unlikely to be driven by reverse causation, broad disease classification, or competing mortality risks and strengthen confidence in the robustness of the primary results.

The clinical implications of these findings extend beyond sleep itself. Long sleep duration may function as an easily obtainable clinical signal of elevated residual risk among adults with heart disease, identifying individuals who may benefit from further evaluation for sleep-disordered breathing, depression, frailty, multimorbidity, medication burden, or declining functional status. Unlike many traditional cardiovascular risk markers, sleep duration can be assessed rapidly, noninvasively, and at virtually no cost across clinical and community settings. As cardiovascular care increasingly emphasizes holistic and lifestyle-based approaches to secondary prevention, routine assessment of sleep duration may offer an additional opportunity to identify patients at elevated risk who might otherwise go unrecognized.

This study has several strengths, including linkage to long‑term mortality follow‑up and adjustment for multiple demographic, behavioral, and clinical covariates. Nevertheless, several limitations should be considered. Sleep duration was self‑reported and measured at a single time point, which may introduce misclassification and may not reflect long‑term patterns. However, self‑reported sleep duration has demonstrated reasonable validity for population‑level surveillance and epidemiologic research. Residual confounding from factors such as depression, obstructive sleep apnea severity, medication use, and cardiovascular disease severity remains possible. In addition, although the study population is heterogeneous and findings were examined in an angina subgroup in sensitivity analyses, angina was not used as part of the primary heart disease case definition. However, findings were robust across multiple sensitivity analyses, including disease-specific cohorts, exclusion of early deaths, competing-risk models, and survey-weighted analyses, supporting the consistency of the observed associations. Finally, our findings are generalizable only to non-institutionalized adult population, and as an observational study, causality cannot be inferred; long sleep duration may represent a consequence rather than a cause of poor health.

Among US adults with heart disease, long sleep duration was consistently associated with higher risks of all-cause, heart-related, and non-heart-related mortality over approximately 15 years of follow-up. These associations persisted across multiple sensitivity analyses and were broadly consistent across demographic subgroups. Although long sleep duration is unlikely to represent a direct causal determinant of mortality, it may serve as a readily accessible clinical indicator of elevated residual risk. Incorporating routine sleep assessment into cardiovascular care may provide a simple and scalable opportunity to enhance risk stratification among adults living with heart disease. Future studies using objective and repeated sleep measures should clarify whether long sleep duration reflects underlying health vulnerability, sleep disorders, or disease progression in adults with established heart disease.

## Supporting information

S1 Table Sensitivity analysis. (DOCX)
