# Supplementary material for: Sleep duration and cause-specific mortality among US adults with heart disease: A NHIS-NDI linkage cohort study
Source: PLoS One. 2026 Nov 13;21(11):e0362657. doi: 10.1371/journal.pone.0362657 (PMC13766240; doi:10.1371/journal.pone.0362657)
Supplement: Supplementary file 1 [file pone.0362657.s001.docx]

# SUPPLEMENTAL MATERIALS

**S1 Table. Sensitivity Analysis**

|  | **All-Cause** | | | **Heart-Related** | | | **Non-Heart-Related** | | |
| --- | --- | --- | --- | --- | --- | --- | --- | --- | --- |
|  | **≤6 hours** | **7-8 hours** | **≥ 9 hours** | **≤6 hours** | **7-8 hours** | **≥ 9 hours** | **≤6 hours** | **7-8 hours** | **≥ 9 hours** |
| **A** | 1.03 (0.91, 1.16) | 1 (ref) | 1.50 (1.29, 1.74) | 0.88 (0.71, 1.10) | 1 (ref) | 1.61 (1.26, 2.05) | 1.10 (0.95, 1.29) | 1 (ref) | 1.44 (1.20, 1.74) |
| **B** | 0.97 (0.86, 1.10) | 1 (ref) | 1.42 (1.22, 1.65) | 0.86 (0.69, 1.07) | 1 (ref) | 1.51 (1.18, 1.94) | 1.04 (0.89, 1.20) | 1 (ref) | 1.37 (1.14, 1.65) |
| **C** | 1.12 (0.95, 1.31) | 1 (ref) | 1.64 (1.35, 1.99) | 1.09 (0.83, 1.42) | 1 (ref) | 1.82 (1.32, 2.50) | 1.14 (0.94, 1.39) | 1 (ref) | 1.55 (1.21, 1.98) |
| **D** | 1.17 (0.98, 1.41) | 1 (ref) | 1.58 (1.27, 1.95) | 1.00 (0.74, 1.36) | 1 (ref) | 1.63 (1.16, 2.29) | 1.29 (1.03, 1.62) | 1 (ref) | 1.55 (1.18, 2.04) |
| **E** | 1.22 (0.96, 1.55) | 1 (ref) | 1.67 (1.23, 2.27) | 1.22 (0.80, 1.84) | 1 (ref) | 1.96 (1.20, 3.20) | 1.21 (0.91, 1.63) | 1 (ref) | 1.49 (1.01, 2.21) |
| **F** | 1.02 (0.87, 1.18) | 1 (ref) | 1.59 (1.35, 1.89) | 0.79 (0.61, 1.03) | 1 (ref) | 1.41 (1.06, 1.89) | 1.15 (0.96, 1.38) | 1 (ref) | 1.70 (1.38, 2.09) |
| **G** |  | 1 (ref) |  | 0.87 (0.71, 1.06) | 1 (ref) | 1.28 (1.02, 1.60) | 1.12 (0.98, 1.29) | 1 (ref) | 1.27 (1.07, 1.52) |
| **H** | 1.06 (0.96, 1.16) | 1 (ref) | 1.77 (1.58, 1.98) | 0.89 (0.76, 1.05) | 1 (ref) | 1.75 (1.47, 2.08) | 1.14 (1.02, 1.28) | 1 (ref) | 1.77 (1.55, 2.02) |
| **I** |  | 1 (ref) |  | 0.87 (0.72, 1.05) | 1 (ref) | 1.46 (1.18, 1.80) | 1.13 (1.00, 1.30) | 1 (ref) | 1.57 (1.32, 1.87) |
| **J** | 1.02 (0.90, 1.16) | 1 (ref) | 1.56 (1.33, 1.82) | 0.93 (0.75, 1.16) | 1 (ref) | 1.59 (1.23, 2.06) | 1.07 (0.91, 1.26) | 1 (ref) | 1.54 (1.27, 1.87) |

A: further adjusting for digestive problems as a confounder (n=3287); B: Reverse Causation analysis, excluding early deaths (first 2 years of follow-up; n=3058); C: restricting to participants with coronary heart disease analysis (n=1310); D: restricting to participants with prior myocardial infarction (n=1003); E: restricting to participants with prior angina (n=567); F: restricting to participants with any other prior heart disease/condition (n=2231); G: Fine-Gray analysis to apply competing-risk models (n=3287); H: survey-weighted sensitivity analysis (unweighted n=3287, weighted n=21188103); I: including cerebrovascular deaths in cause-specific deaths (n=3287); and J: excluding cancer survivors (n = 2719).
